# A framework for exploring associations between biomedical terms in PubMed

**DOI:** 10.18632/oncotarget.21532

**Published:** 2017-10-05

**Authors:** Haixiu Yang, Lingling Zhao, Ying Zhang, Hong Ju, Dong Wang, Yang Hu, Jun Zhang, Liang Cheng

**Affiliations:** ^1^ College of Bioinformatics Science and Technology, Harbin Medical University, Harbin 150081, PR China; ^2^ School of Computer Science and Technology, Harbin Institute of Technology, Harbin 150001, PR China; ^3^ Department of Rehabilitation and Pharmacy, Heilongjiang Province Land Reclamation Headquarters General Hospital, Harbin 150088, PR China; ^4^ Department of Information Engineering, Heilongjiang Biological Science and Technology Career Academy, Harbin 150001, PR China; ^5^ Department of Academic Research, Heilongjiang University of Science & Technology, Harbin 150022, PR China; ^6^ School of Life Science and Technology, Harbin Institute of Technology, Harbin 150001, PR China

**Keywords:** co-occurrence relationship, text mining, framework, term association

## Abstract

Co-occurrence relationships in PubMed between terms accelerate the recognition of term associations. The lack of manually curated relationships in vocabularies and the rapid increase of biomedical literatures highlight the importance of co-occurrence relationships. Here we proposed a framework to explore term associations based on a standard procedure that comprises multiple tools of text mining and relationship degree calculation methods. The text of PubMed were segmented into sentences by Apache OpenNLP first, and then terms of sentences were recognized by MGREP. After that two terms occurring in a common sentence were identified as a co-occurrence relationship. The relationship degree is then calculated using Normalized MEDLINE Distance (NMD) or relationship-scaled score (RSS) method. The framework was utilized in exploring associations between terms of Gene Ontology (GO) and Disease Ontology (DO) based on co-occurrence relationship. Results show that pairs of terms with more co-occurrence relationships indicate shared more semantic relationships of ontology and genes. The identified association terms based on co-occurrence relationships were applied in constructing a disease association network (DAN). The small giant component confirms with the observation that diseases in the same class have more linkage than diseases in different classes.

## INTRODUCTION

A large number of biomedical terms occurs with the rapid development of researches and the increasement of the literatures. Associations between these terms play important roles in exploring literature and linking term-related molecules [1, 2]. e.g. Medical Subject Headings (MeSH) [3] classified biomedical terms in 16 categories based on set inclusion relationship between terms for easing store and retrieve literatures [3]. After applying annotating the function of molecules, the advantages of associations between terms gradually appears. Recent studies have utilized these term associations in constructing functional similarity network of non-coding RNAs (ncRNAs) [4, 5], predicting novel disease related ncRNAs [5], and so on.

Term associations can often be reflected by the manually curated relationships between terms in ontologies or their annotating genes. Terms of a domain are collected as a biomedical ontology or a category of an ontology. For example, terms of biological process (BP), molecular function (MF) and cell component (CC) were organized in three categories of Gene Ontology (GO) [6]. ‘IS_A’ relationships between terms of sub-categories of GO form a directed acyclic graph (DAG). The DAG was frequently used in calculating similarity of pair-wise terms, and the high similarity scores represent the association between terms [7–9]. Currently, Wang et al.’s method [4, 10] was validated suitable in biomedical ontologies, such as GO, disease ontology (DO) [11], and so on. Because no relationships between different terms across ontologies exist, it is not easy to explore associations between terms across ontologies based on the DAG of ontology completely. Fortunately, the functional annotations of genes help for this. To explore associations between terms across ontologies, three methods involving Vector Space Model (VSM) [12], Association Rule Mining (ASR) [13], and Cross-Category Gene Ontology Measurement (CroGO) methods [14] were designed.

Two terms occurs in common sentence, abstract, documents of a literature is deemed as co-occurrence relationships. The co-occurrence relationships occur widely in common literatures of PubMed. In comparison with DAG and term gene relationship, these relationships were easier to be ignored for exploring term associations. Currently, four methods involving Normalized MEDLINE Distance (NMD) [15], relationship-scaled score (RSS) [16], extensional Mutual Information (EMI) [17], and adjusted RSS based on information content (ARSSIC) methods [18] were presented based on co-occurrence relationships. NMD method is based on Normalized Google Distance (NGD) method [19], which is implemented in google search engine for calculating the relationship degree of two search terms based on their co-occurrence relationships. RSS and EMI methods are designed based on MI (Mutual Information) [20] to rank relativities between terms in literature. Compared with other three methods, ARSSIC method incorporates DAG of ontology and the co-occurrence relationships.

Although multiple methods have been presented for exploring associations between terms based on their co-occurrence relationships in PubMed, two issues limit the application of these methods. One issue is that two terms occurring in common abstract of literature were extracted for co-occurrence relationships. Obviously, two terms occurring in common sentence of abstract should be more likely to have potential linkages. The other issue is that no procedure was presented to extract co-occurrence relationships between terms from literature. To solve these two issues, here we designed a framework to annotate literature with biomedical ontologies and explore term associations. First, the abstract of literatures were segmented into sentences. And then the terms of sentences were mapped to biomedical ontology terms. Finally, the similarity based on co-occurrence relationships was calculated based on existing methods to reflect the relationship degree of pair-wise terms.

## RESULTS

### Co-occurrence relationships between GO and DO terms in PubMed

Terms with co-occurrence relationships were extracted based on our framework in February 2017. After removing the annotations without more than two terms in a sentence, 2,057 CC terms (52.65%–2057/3907), 3,708 MF terms (37.12%–3708/9988), 9,588 BP terms (33.95%–9588/28245), and 5,291 DO terms (76.46%–5291/6920) in 15,922,610 sentences of abstracts were left. Totally, a larger number of BP terms are related with other terms, and the highest proportion of disease terms is analyzed with other terms. It is rational that disease domain deserves more attention.

**Figure 1:**
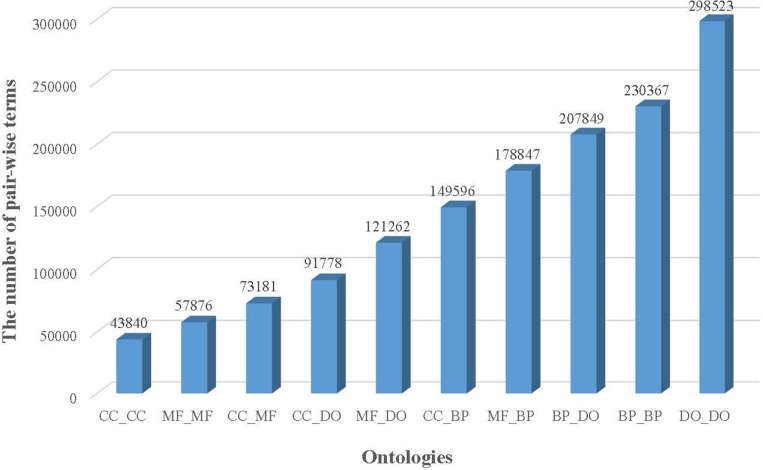
The number of pair-wise terms of ontologies with co-occurrence relationships

The more detailed annotation results are shown in Figure 1. In all 1,453,119 pairs of terms with co-occurrence relationships were obtained. Among them, 43,840 CC term pairs, 57,876 MF term pairs, 230,367 BP term pairs, 298,523 DO term pairs have co-occurrence relationships, and larger number of relationships across ontologies were extracted. In comparison with the number of ‘IS_A’ relationships from ontology, more relationships exist in PubMed. e.g. almost 8 times the number of relationships between CC terms (7.80–43840/5618) occur in PubMed. Therefore, it is very important to access co-occurrence relationships to aid to explore the association between terms of an ontology. Since few number of manually curated relationships were provided between terms across ontologies, co-occurrence relationship plays important roles in exploring term associations across ontologies across ontologies.

### Related terms based on co-occurrence relationships indicate shared semantic relationships of ontology

In order to assess the performance of term relativity based on co-occurrence relationships in PubMed, we compared the correlation between term relativity with structure-based term similarity. The term similarity based on ontology performs well and is widely used in biomedical domain as best of our knowledge. Thus, the correlation can show the performance of term relativity. Because RSS and NMD methods [15, 16] are two typical methods based on MI and NGD respectively, these two methods are used for calculating relative degree of pair-wise terms here. Wang’s method completely depends on the structure of ontology. Therefore, Wang’s method was utilized for calculating term similarity of BP, MF, CC and DO.

**Figure 2:**
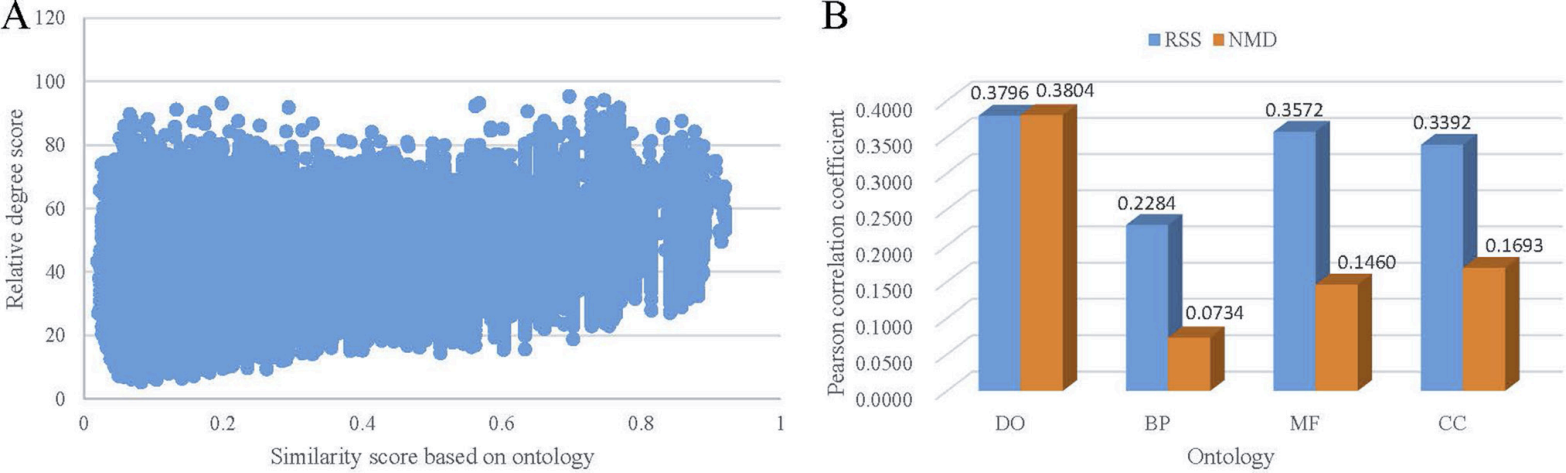
The correlation between co-occurrence based term relativity and structure-based term similarity (**A**) The distribution of the term similarity by Wang’s method. (**B**) Pearson correlation coefficient between relative degree score and similarity score.

Figure 2A shows the scatter plot of the distribution of RSS score and similarity score of DO term pairs. The Pearson correlation coefficient is 0.3796 (*p* < 2.2e–16). The validation result demonstrates that the related terms based on co-occurrence relationships achieve high similarity scores based on ontology. The same comparison was practiced on three sub-categories of GO. And the Pearson correlation coefficient is 0.2284, 0.3572 and 0.3392 for BP, MF and CC term pairs, respectively (Figure 2B). These results indicate the performance of RSS score is stable for exploring term associations of different ontologies. In comparison with three categories of GO, the highest proportion of DO terms have co-occurrence relationships in PubMed. Correspondingly, the highest correlation was obtained between RSS relative score and similarity score of DO term pairs. On the contrary, the lowest proportion of BP terms have co-occurrence relationships, and the lowest correlation was obtained between RSS relative score and similarity score of BP term pairs.

We also compared the correlation between NMD score and similarity score of ontology term pairs. The results are shown in Figure 2B. The Pearson correlation coefficient is 0.3804, 0.0734, 0.1460 and 0.1693 for DO, BP, MF and CC term pairs, respectively. In all, the results based on NMD score are in accordance with the results based on RSS score. And the correlation based on NMD score is slightly less than the correlation based on RSS score. Since both RSS and NMD methods are based on co-occurrence relationships, the reliability of the relative terms based on PubMed are further validated. Considering they are two different types of methods, the results in Figure 2 show that the performance of RSS method is a litter better than NMD method.

### Related terms based on co-occurrence relationships indicate shared genes

Semantic relationships between different terms mainly occur in single ontology. Hence, it is not easy to assess the co-occurrence relationships based on structure of existing ontologies. Since DO and GO terms are frequently used for annotating the function of genes [9, 21], the similarity between terms across DO and GO terms can be calculated using ASR method [13] based on their annotated genes. In order to assess the performance of term relativity across ontologies, we compared the correlation between term relativity with gene-based term similarity.

**Figure 3:**
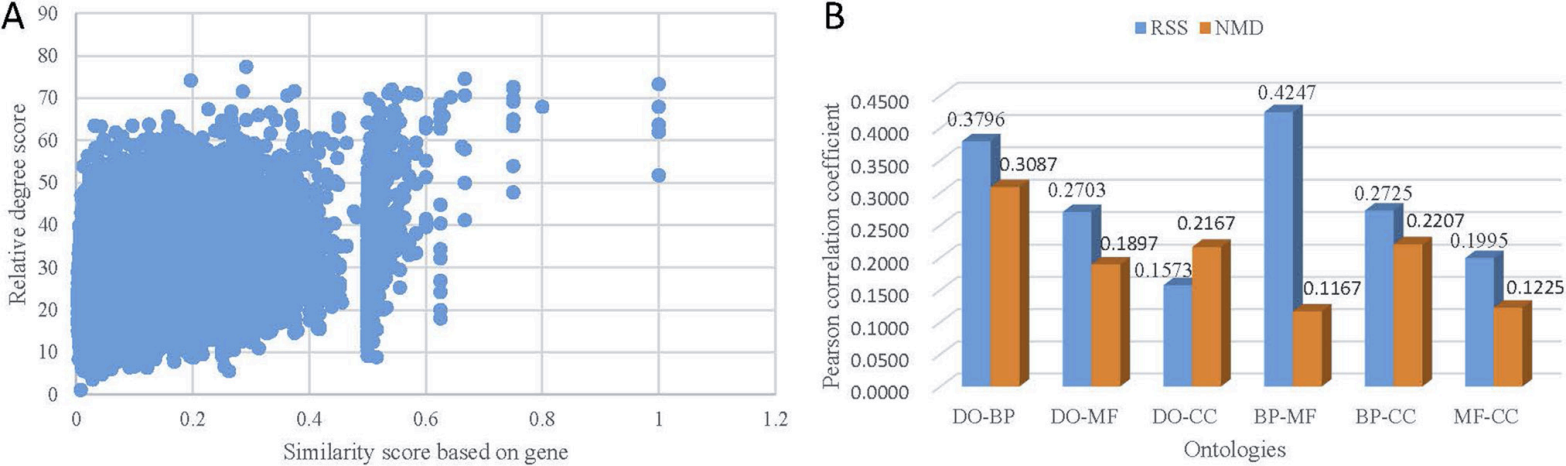
The correlation between co-occurrence based term relativity and gene-based term similarity (**A**) The distribution of the term similarity by ASR method. (**B**) Pearson correlation coefficient between relative degree score and ASR score.

The scatter plot of the distribution of RSS score and ASR score of DO-BP term pairs is shown in Figure 3A (Pearson correlation, g2 = 0.3796 *p* < 2.2e–16). This result demonstrates that the related terms across ontologies based on co-occurrence relationships achieve high similarity scores based on their annotated genes. The further validation is performed on DO-MF, DO-CC, BP-MF, BP-CC, and MF-CC term pairs, the corresponding Pearson correlation coefficient is 0.2703, 0.1573, 0.4247, 0.2725, and 0.1995, respectively. It show that related terms across ontologies based on co-occurrence relationships indicate shared genes. In consideration of the above results on single ontology, all of these results in Figure 3 verify that the RSS score based on co-occurrence relationship is suitable for exploring associated term pairs.

The NMD score of terms across ontologies was also compared with ASR score. The Pearson correlation coefficient is 0.3087, 0.1897, 0.2167, 0.1167, 0.2207, and 0.1225 for DO-BP, DO-MF, DO-CC, BP-MF, BP-CC, and MF-CC term pairs, respectively (Figure 3B). On the whole, both NMD score and RSS score achieve high correlation between terms across GO and DO. Among them, the correlation based on NMD score of DO-CC term pairs is slightly higher than that based on RSS score. And the correlation based on NMD score of DO-BP, DO-MF, BP-MF, BP-CC, and MF-CC term pairs is lower than based on RSS score. These results in Figure 3 are consistent with results in Figure 3, which validates that RSS method achieves better performance than NMD method.

### Case study of constructing disease association network

In order to show the utilization of associated terms in PubMed, we constructed a term association network. In the network, a node represents a term, and two nodes are linked by an edge if the relative score of them is equal or larger than a threshold of relative score. Considering the highest proportion of DO terms has co-occurrence relationships in PubMed, a disease association network (DAN) was established based on the related disease pairs. In addition, because RSS method accesses higher performance than NMD method, RSS score between DO terms was calculated for this purpose.

**Figure 4:**
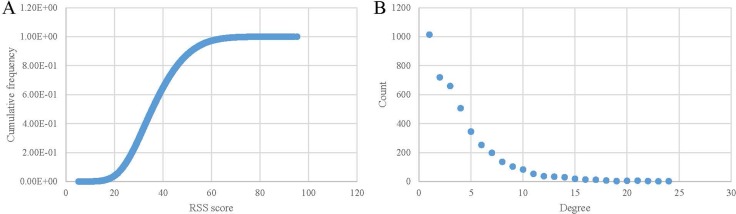
Constructing and characteristics of the disease association network (**A**) Cumulative distribution of the edges between diseases when using various similarity thresholds. (**B**) Degree distribution for diseases in the DAN.

The threshold of relative score is accessed based on the distribution of the RSS scores of all DO term pairs, which is shown in Figure 4A. The number of links dramatically decreases when the threshold increases from 20 to 60. It means that the edges with score lower than 20 and higher than 60 are seldom. According to the previous validation, term pairs with the larger score can be more likely to be associated with each other. In addition, when the threshold is equal or larger than 60, the link numbers remain relatively stable. Hence, we choose 60 as the threshold to set up DAN.

8,531 associations between 4,241 diseases were utilized in constructing the DAN (Supplementary Figure 1). We then analyzed the degree of the network. Figure 4B demonstrates the histogram of the number of diseases in the network linked with the other diseases. 1,014 (23.91%–1014/4241) diseases are connected with only one disease. In comparison, 2,508 (59.14%–2508/4241) diseases are adjacent with three or more diseases. ‘eccrine porocarcinoma (DOID:7566)’ and ‘osteochondrodysplasia (DOID:2256)’ are the hub diseases in the network, which contains the most number of associated diseases (24). According to the previous study [22], diseases of Online Mendelian inheritance in man (OMIM) [23] are categorized into 22 classes. And diseases in the same class have more linkage than diseases in different classes. Therefore, the giant component of DAN should be smaller than the random network. Actually, the giant component of our network contains 3,885 diseases (9.999e-05 – 1/10001), which is the least number of diseases in the DAN compared to 10,000 random networks. The further examination of the degree distribution of the diseases in the DAN revealed a power-law distribution with R^2^ = 0.896 (Figure 4B). This indicated that the network displayed scale-free characteristics like many other biological networks [22, 24, 25].

## DISCUSSION

In this article, we designed a framework to explore associations between biomedical terms in PubMed. Tools for extracting co-occurrence relationships and methods for calculating relationship degree are two main issues for this purpose. Unlike the previous methods in describing the co-occurrence relationship, we define it as the occurrence of two terms in common sentence. Obviously, our co-occurrence relationship shows stronger relationship degree than that of the occurrence of two terms in common document. To extract the co-occurrence relationship, text of PubMed should be segmented into sentences by Apache OpenNLP first, and then annotated by term recognition tool MGREP. Subsequently, the relationship degree can be calculated by existing methods based on co-occurrence relationships.

The framework was utilized for establishing the association between terms of DO and GO. As a result, a large number of relationships between ontology terms were extracted from PubMed. Experiments validated that related terms based on co-occurrence relationships indicate shared semantic relationships of ontology and shared genes. The framework was easily to be extended for exploring terms of other biomedical ontologies as expected. Therefore, our framework can be regarded as a complement to the manually curated relationships between terms. The case study of established DAN shows an application of the identified associated terms. The network confirms with the observation that diseases in the same class have more linkage than diseases in different classes. This further validates the reliability of the term associations. The identified term associations can also be utilized for constructing network for BP, MF, CC and other ontologies.

The relative score based on co-occurrence is one type of relationship between terms. There are also other types of relationships between terms, such as structure-based and gene-based. Therefore, the Pearson correlation coefficient among these different associations cannot decide the performance of each type of relationships among terms. Here, we investigated correlations among different types of relationships between terms to illustrate their associations.

## MATERIALS AND METHODS

### Tools

Two tools involving Apache OpenNLP [26] and MGREP [27] are used for sentence segmentation and term recognition. Apache OpenNLP is an open source project for natural language processing (NLP) tasks. It contains a tool named OpenNLP Sentence Detector for sentence segmentation. The tool can detect whether a punctuation character marks the end of a sentence or not. In this sense a sentence is defined as the longest white space trimmed character sequence between two punctuation marks. MGREP is a NLP tool for mapping medical free text to formal medical terms [27]. Because it implements a novel radix-tree-based data structure that enables fast and efficient matching of text against a set of dictionary terms, the tool performs slightly better in terms of accuracy and speed typical tool for term recognition [28]. MGREP was utilized as a mapping tool by Open Biomedical Annotator (OBA) [29], which automates the process of extracting terms from text that are available on the web. However, it is not an easy way for us to extract terms of text in PubMed. Thus, we contact with Dai et al to get the MGREP binary and run it locally.

### Vocabularies and literature set and ontology annotations

Two vocabularies including GO and DO were downloaded from open source repositories (Table 1), which provided manually curated ‘IS_A’ relationships between terms. Currently, a total of 12,174 ‘IS_A’ relationships between 9,988 MF terms, 54,502 ‘IS_A’ relationships between 28,245 BP terms, 5,618 ‘IS_A’ relationships between 3,907 CC terms, and 7,124 ‘IS_A’ relationships of 6,920 DO terms were included in these ontologies. Literature set was obtained from PubMed (Table 1). Currently, it contains tens of millions of literatures which were documented in XML format.

**Table 1:** Data sources

Data source	Web site
GO	http://geneontology.org/page/download-ontology
GOA	http://geneontology.org/gene-associations/gene_association.goa_ref_human.gz
DO	http://disease-ontology.org/
DOA	http://www.bio-annotation.cn/gene2function/
PubMed	ftp://ftp.ncbi.nlm.nih.gov/pubmed/baseline

GO annotations (GOA) [30] of human genes were accessed from GO Consortium (Table 1). After removing annotations of inferred from electronic annotation (IEA), 38,205 annotations between 3,217 MF terms and 14,435 human genes, 94,779 annotations between 9,032 BP terms and 14,272 human genes, and 46,968 annotations between 1,323 CC terms and 14,625 human genes were obtained. DO Annotations (DOA) [31] were sourced from the annotations of GeneRIF [32]. After removing the duplication records, 98,008 associations between 2,576 diseases and 9,991 genes were obtained.

### The framework for exploring term associations

**Figure 5:**
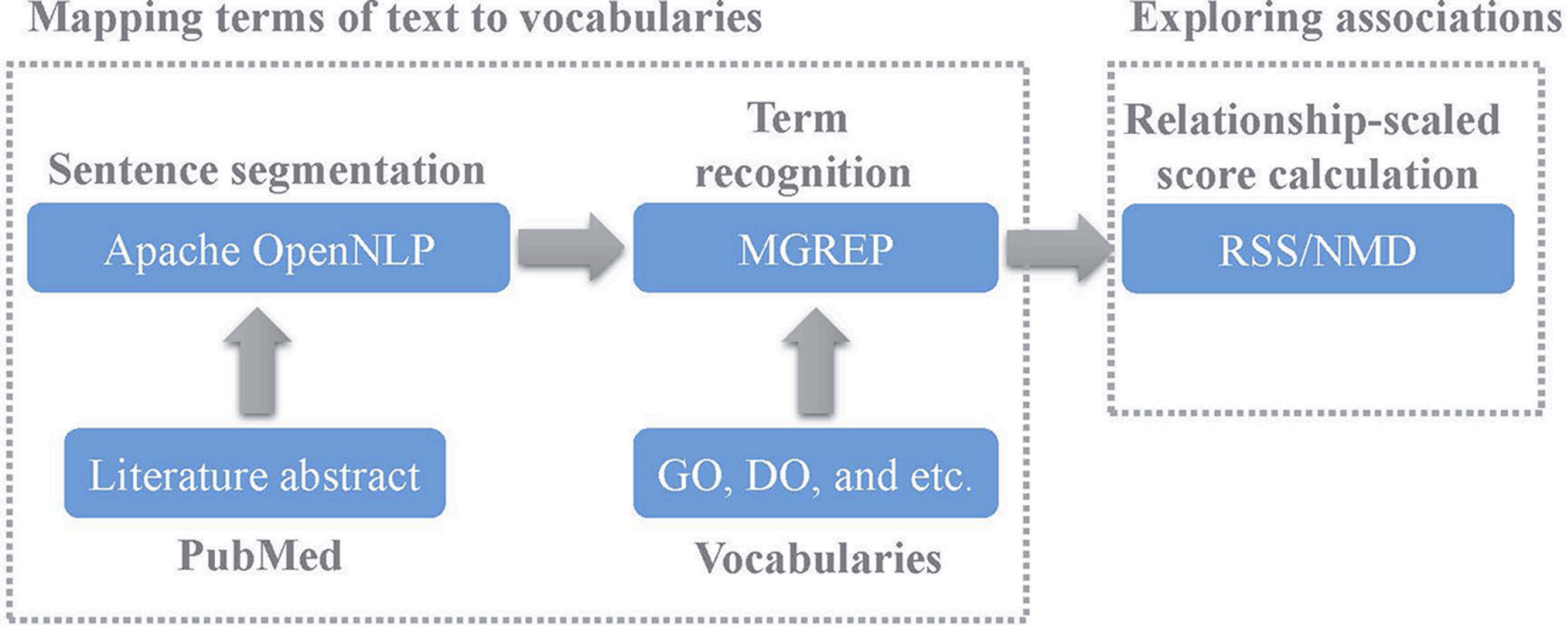
The framework for exploring term associations

Here we extracted the terms from abstract of literature of PubMed and established the associations between these terms based on their co-occurrence relationships. The framework for exploring term associations is shown in Figure 5. The details is described as following.

### Mapping terms of PubMed to vocabularies

First, a script was implemented for extracting abstracts of literatures from PubMed. Next, the abstract of each literature was segmented into several sentences based on OpenNLP. Then, the terms of sentences were mapped to GO and DO using MGREP. As a result, the terms with co-occurrence relationships in the common sentences were extracted from PubMed. Certainly the terms of sentence could be mapped to other biomedical ontologies as expected.

### Exploring associations

The co-occurrence degrees between terms were calculated based on RSS or NMD methods. In theory the four methods involving RSS, EMI, NMD, ARSSIC methods could be selected as expected. Because ARSSIC method incorporate semantic relationships between terms, it is not suitable for exploring term associations based on co-occurrence relationships completely. Since both RSS and EMI methods are based on MI, the performance of these two methods are similar. Considering of validating the term associations based on co-occurrence relationships is independent of the different types of methods, here we selected RSS and NMD methods.

We define occurrence value (OV) to represent how much a term *t* related to a sentence *s* as following:

$$OV\left(t,s\right)=\{\begin{array}{cc} 1 & \mathrm{if}\;t\;\text{occurs in}\;s.\\ 0 & \mathrm{if}\;t\;\text{doesn’t occur in}\;s. \end{array}$$ (1)

Based on equation 1, the OV of *t* in s is zero if *t* doesn’t occur in s. Consequently, a co-occurrence value (COV) between a pair of terms *t1* and *t2* is defined as follows:

$$COV\left(t_{1},t_{2}\right)=\sum_{i=0}^{n}\left(OV\left(t_{1},s_{i}\right)\cdot OV\left(t_{2},s_{i}\right)\right),$$ (2)

where *n* represents the total number of co-occurring literature of *t1* and *t2*, and *si* indicates the *i*th sentence. The COV is then utilized to calculate and normalize RSS in equation 3 and 4.

$$RSS\left(t_{1},t_{2}\right)=\log_{10}\left(\frac{COV\left(t_{1},t_{2}\right)}{COV\left(t_{1},t_{1}\right)\cdot COV\left(t_{2},t_{2}\right)}\right),$$ (3)

$$RSS_{N}\left(t_{1},t_{2}\right)=1+\frac{99*\left(RSS\left(t_{1},t_{2}\right)-RSS_{\min}\right)}{\left(RSS_{\max}-RSS_{\min}\right)},$$ (4)

where RSS(t_1_, t_2_) and RSS_N_(t_1_, t_2_) represent the RSS and the normalized RSS between *t1* and *t2*, respectively; RSS_max_ and RSS_min_ represent the maximum and the minimum RSS, respectively. As a result, RSS score of term pairs ranges from 1 to 100.

Based on NMD method, the distance between term *t1* and *t2* is defined in Equation 5.

$$NMD\left(t_{1},t_{2}\right)=\frac{\max\left(\log\sum_{i=1}^{M}OV\left(t_{1},l\right),\log\sum_{i=1}^{M}OV\left(t_{2},l\right)\right)-COV\left(t_{1},t_{2}\right)}{\log\left|M\right|-\min\left(\log\sum_{i=1}^{M}OV\left(t_{1},l\right),\log\sum_{i=1}^{M}OV\left(t_{2},l\right)\right)},$$ (5)

where *M* is the total number of literatures. Because the relationship degree is inversely proportional to the term distance, the relationship degree between *t1* and *t2* based on NMD method is defined in Equation 6.

$$Sim_{NMD}\left(t_{1},t_{2}\right)=\frac{\max\left(\log\sum_{i=1}^{M}OV\left(t_{1},l\right),\log\sum_{i=1}^{M}OV\left(t_{2},l\right)\right)-COV\left(t_{1},t_{2}\right)}{\log\left|M\right|-\min\left(\log\sum_{i=1}^{M}OV\left(t_{1},l\right),\log\sum_{i=1}^{M}OV\left(t_{2},l\right)\right)}.$$ (6)

## SUPPLEMENTARY MATERIALS FIGURE


