# Supplementary material for: A framework for exploring associations between biomedical terms in PubMed
Source: Oncotarget. 2017 Oct 5;8(61):103100–7. doi: 10.18632/oncotarget.21532 (PMC5732714; doi:10.18632/oncotarget.21532)
Supplement: Supplementary file 1 [file oncotarget-08-103100-s001.pdf]

A framework for exploring associations between biomedical terms in PubMed

SUPPLEMENTARY MATERIALS

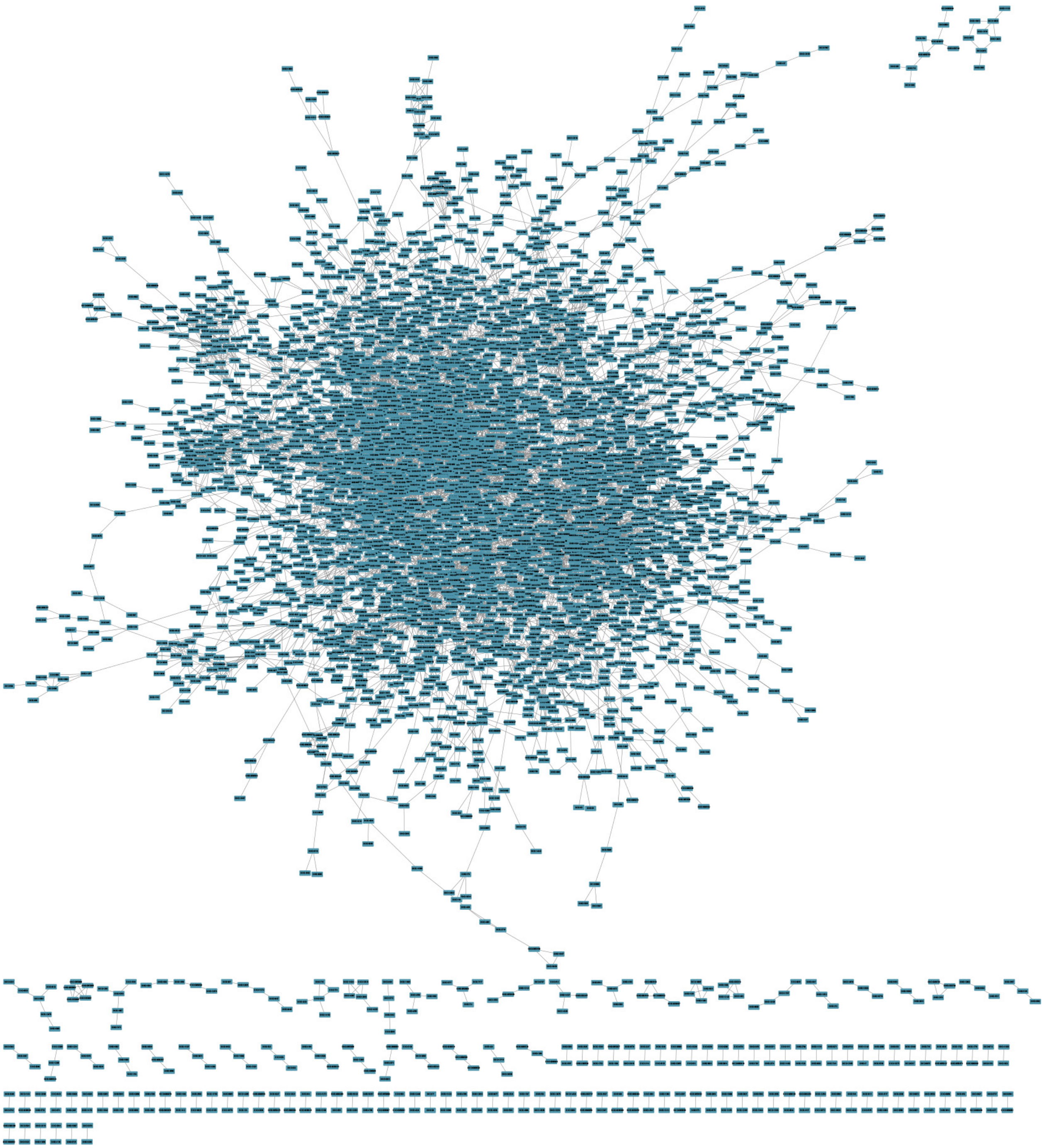

Supplementary Figure 1: The disease association network based on PubMed.
